# Finding the best combination of autochthonous microorganisms with the most effective biosorption ability for heavy metals removal from wastewater

**DOI:** 10.3389/fmicb.2022.1017372

**Published:** 2022-10-06

**Authors:** Violeta Jakovljević, Sandra Grujić, Zoran Simić, Aleksandar Ostojić, Ivana Radojević

**Affiliations:** ^1^Department of Natural-Mathematic Sciences, State University of Novi Pazar, Novi Pazar, Serbia; ^2^Department of Biology and Ecology, Faculty of Science, University of Kragujevac, Kragujevac, Serbia; ^3^Department of Chemistry, Faculty of Science, University of Kragujevac, Kragujevac, Serbia

**Keywords:** autochthonous microorganisms, biofilms, biosorption, cocultures, heavy metals, wastewater

## Abstract

The presence of heavy metals (HMs) in the environment represents a serious environmental problem. In this regard, this work was conceived with the aim of finding, among indigenous microorganisms, the species and their combinations with the best biosorption activity for the following HMs: zinc, lead, cadmium, copper, and nickel. The experiment was carried out in several steps: (1) isolation and identification of microbial strains from the Central Effluent Treatment Plant’s wastewater; (2) studying the interaction of microorganisms and the ability to form biofilms in 96-well plates; (3) testing the resistance of biofilms to HMs; (4) testing the growth of biofilms on AMB media carriers in the presence of HMS; and (5) biosorption assay. The selected strains used in this study were: *Enterobacter cloacae, Klebsiella oxytoca, Serratia odorifera*, and *Saccharomyces cerevisiae*. The best biofilm producers in control medium were *K. oxytoca*/*S. odorifera* (KS), followed by *K. oxytoca*/*S. odorifera/S. cerevisiae* (KSC), and *E. cloacae/K. oxytoca*/*S. odorifera* (EKS) after 10 days of incubation. Mixed cultures composed of three species showed the highest resistance to the presence of all tested metals. The best biosorption capacity was shown by KSC for Cu^2+^ (99.18%), followed by EKS for Pb^2+^ (99.14%) and Cd^2+^ (99.03%), *K. oxytoca* for Ni^2+^ (98.47%), and *E. cloacae* for Zn^2+^ (98.06%). This research offers a novel approach to using mixed biofilms for heavy metal removal processes as well as its potential application in the bioremediation of wastewater.

## Introduction

According to the definition, heavy metals (HMs) are metals having a specific weight greater than 5.0 g/cm^3^. Rapid industrial and technological advancement has increased HMs water contamination (Tchounwou et al., 2012). Toxic HMs, including chromium (Cr^3+^), lead (Pb^2+^), zinc (Zn^2+^), arsenic (As^3+^), copper (Cu^2+^), nickel (Ni^2+^), cobalt (Co^2+^), cadmium (Cd^2+^), and mercury (Hg^2+^), are persistent in the environment and tend to bioaccumulate in living beings, resulting in a wide range of diseases (Ghosh et al., 2021). The HMs’ toxicity occurs even at very low values of 1.0–10 mg/l. As the quantity of HMs in the environment has risen, numerous physical and chemical procedures for their removal have been developed and are continually being improved (Renu and Singh, 2017; Zahir et al., 2021; Azam et al., 2022). These approaches, however, have limited success. Some metal removal procedures, for example, have been shown to be inefficient in low metal ion concentration settings (>100 mg/l) (Díaz et al., 2022). Furthermore, the high expense of the equipment and process that results in the formation of intermediary chemicals with possibly more hazardous effects than the initial pollutants is a constraint. As a result, biosorption has piqued the curiosity of many researchers in recent decades (Pham et al., 2022).

Recently, research has focused on the use of indigenous microorganisms in environmental bioremediation processes. A contaminated environment has decreased variety while increasing the “richness” of select groups that have adapted to living in the presence of contaminants (Roling et al., 2002). Among these creatures, microorganisms and certain fungi predominate (Dusengemungu et al., 2020; Younas et al., 2021). Because of the large number of HM pollutants prevalent in the environment and the availability of many different types of microorganisms, it is not unexpected that efforts are being made to isolate and identify strains from polluted settings that might be utilized for HM bioremediation by diverse mechanisms at extracellular and intracellular levels. Biosorption has been demonstrated to successfully remove a wide range of HMs from aqueous solutions, including very hazardous metal ions like Cd^2+^, Cr^3+^, Pb^2+^, Hg^2+^, and As^3+^ (Saba et al., 2019). It is a contemporary, low-cost technology that employs organisms such as bacteria, algae, yeasts, filamentous fungus, and others (He and Chen, 2014; Javanbakht et al., 2014; Redha, 2020). *Klebsiella aerogenes* bacterial cells (Gutnick and Bach, 2000), as well as *S. cerevisiae* (Cojocaru et al., 2009) and *Candida rugosa* (Basak et al., 2014) yeasts, have shown promising HMs removal results to date. *Bacillus tropicus* MCCC 1A01406 was recently discovered and described as being resistant to multiple heavy metals, including Pb^2+^, Cr^3+^, Cd^2+^, Cu^2+^, Ni^2+^, Fe^2+^, Zn^2+^, and Co^2+^ (Saha et al., 2022).

Microbial biofilms are thought to be the most common type of microbial life that arose as a response to harsh environmental circumstances, offering an ecological survival benefit (Hall-Stoodley et al., 2004). Individual biofilms have been widely studied in the process of HMs removal in recent years (Harrison et al., 2006; Singh et al., 2006; Grujić et al., 2017a, 2018). However, research on HMs removal by mixed biofilms is limited (Buzejić et al., 2016; Grujić et al., 2017b).

Several studies have found the influence of biofilms, or planktonic microorganisms isolated from polluted environments, on HMs bioremediation (Golby et al., 2014; Frankel et al., 2016a,b), but none of the studies refer to specific combinations of individually separated microorganisms. As a result, the goal of this research is to isolate and identify microorganisms capable of forming biofilms from wastewater and to choose the “perfect combination” that produces a mixed-species biofilm that is most successful in HMs removal.

## Materials and methods

### Microorganisms: Isolation, identification, and growth conditions

Microorganisms were isolated from the wastewater of the Central Effluent Treatment Plant’s wastewater (Kragujevac, Serbia). The contents of the bioreactor and recirculation pool were collected in sterile plastic bottles (April 2016) and delivered to the microbiology laboratory at the Faculty of Science in Kragujevac, where microbe isolation and identification were carried out. The colonies that were most present in the examined water samples were isolated. Pure cultures were produced by screening the chosen isolates using the exhaustion method. After 24-h incubation of pure cultures on a nutrient medium at temperatures of 26 and 37°C, the morphological properties (shape, presence of spores, Gram staining) were examined using a light microscope (Olympus, U-RFLT-T, GmbH, Germany). The bacteria were isolated using nutrient agar, differential, and selective substrates. Identification of bacterial isolates was performed using standard morphological and biochemical methods (Reddy et al., 2007). The identification of isolated enterobacteria was confirmed using the commercial test for the identification of enterobacteria API 20E (BioMérieux). Samples were prepared according to the manufacturer’s instructions. For the isolation of yeast, tryptic soybean agar (TSA) with streptomycin and a pH of 6.5–6.8 was used. The identification of pure cultures was carried out at the unit for Algology, Mycology, and Lichenology, at the Institute of Biology and Ecology, Faculty of Science in Kragujevac, based on morphological characteristics and the application of the fungal identification key. The identification was confirmed using the biochemical API 20C AUX test (BioMérieux). The identification was confirmed using the biochemical API 20C AUX test (BioMérieux).

In polystyrene microtiter plates, the discovered bacteria were tested for their capacity to produce single and mixed biofilms in the absence and presence of chosen HMs. The bacteria that produced the biofilm effectively were also employed to assess their capacity to create single and mixed biofilms on AMB Media (Assisting Moving Bed Media) carriers and remove HMs.

Tryptic soybean broth (TSB, MossHemoss) (Harrison et al., 2006) was used to analyze bacteria and yeasts. The direct colony approach was used to create a microorganism suspension. The turbidity of the suspension was adjusted using a densitometer (DEN-1, Biosan, Latvia), McFarland 1.0, to equate to 10^8^ CFU/ml for bacteria and 10^6^ CFU/ml for yeasts (Grujić et al., 2017b).

### Preparation of HM solutions

To assess the metal resistance of the produced biofilms, Pb^2+^, Zn^2+^, Cd^2+^, Cu^2+^, and Ni^2+^ salts were generated from Pb(NO_3_)_2_, ZnSO_4_, CdSO_4_, CuSO_4_, and NiSO_4_ salts (Sigma-Aldrich, St. Louis, MO, USA). No more than 60 min before usage, working solutions were produced in TSB medium from stock solutions. Based on the previous research (Basak et al., 2014) and the preliminary test, a concentration range of 100,000–781 μg/ml was chosen (Buzejić et al., 2016).

### Testing the resistance of planktonic cells and biofilms

The resistance of planktonic cells and their biofilms to the influence of HMs was examined by the Minimum Biofilm Eradication Concentration-High Throughput Plates (MBEC™-HTP, BioProducts) according to Ceri et al. (1999). Two hundred microliters of medium and 20 μl of microorganism suspension were added to each well of the plate, which corresponded to the 96-peg lid. After a period of incubation at 26°C, the planktonic cells in the nutrient medium, as well as the biofilm formed on the pegs, were used to test the resistance in the presence of different concentrations of HMs. The lid with the pegs and the formed biofilms were transferred to the plate with the tested substances. Some of the cells, during the incubation period, will not enter into the formation the biofilm. These cells retain their planktonic phenotype and thus are exposed to the influence of the tested substances. The growth that is recognized as a blur shows that the plankton cells have survived testing. In this challenge plate, the minimum inhibitory concentration (MIC) and the minimal lethal concentration (MLC) are detected by the absence of turbidity using an ELISA reader at 650 nm (Rayito, China).
After the exposure time of the transferred biofilm to the tested substances (24 h and 48 h for bacteria, 48 h and 72 h for yeasts), the peg lid was removed and washed with 0.9% sterile saline, after which the lid was transferred to a new fresh medium (200 μl per well). The plate was exposed to ultrasonic waves (Aquasonic 250 HT Ultrasonic Cleaner, VWR International, Radnor, PA, USA), which removed the biofilm from the pegs into each well. The minimum biofilm eradication concentration (MBEC) of biofilms was determined by spectrophotometric readings on the ELISA reader, OD_650_.

### Biofilm formation and HM resistance of tested biofilms and quantification

The formation of biofilms and the degree of resistance to the tested HM were confirmed in polystyrene microtiter plates with 96 wells (SARSTEDT, Belgrade) according to the described method by Adam et al. (2002) with certain modifications. Plates were filled with 100 μl of medium and 10 μl of microorganism suspension. The plates were then placed in an incubator at 26°C for 24 h for bacteria, 48 h for yeasts, and mixed biofilms. After the incubation period, the tested solutions of HM were added to the formed biofilms and their influence was monitored (the same conditions as incubation). A modified crystal violet (CV) assay with particular modifications (Almeida et al., 2011) was used for the biofilm biomass determination. After incubation, the content from the plates was removed and 50 μl of methanol (98% v/v) was added and incubated again for about 15 min. Before drying at room temperature, it was necessary to remove the methanol content. Then, 50 μl of CV was added for 5 min and washed three times with distilled water, and then 100 μl of glacial acetic acid (33% v/v) was added. The minimum biofilm inhibitory concentration (MBIC) and minimum biofilm eradication concentration (MBEC) were determined by reading the optical density (OD_570_) using a microplate reader (Rayito, China). All measures were made in duplicate, and the MICs and MBECs were constant.

### Determination of biosorption potential of single and mixed-biofilms

#### Experimental setup for the removal of metal ions

The main role of AMB Media carriers (Assisting Moving Bad Media) is to increase the active surface area occupied by microorganisms. The carriers used in this study are made of high density polyethylene (*q* = 1 g/cm^3^) and had the following sizes: outer diameter of 12 mm, length of 13 mm, usable internal surfaces of 500 m^2^/m^3^ and free areas of 85% (Jakovljević et al., 2022).

AMB carriers, pre-sterilized by autoclaving, were placed in 6 well plates. 10 ml TSB broth (diluted with a sterile distilled water ratio of 1:3) was added to each hole of the plate (Beściak and Surmacz-Górska, 2011). The plate was inoculated by adding 100 μl of microbial suspension and incubated at 25°C. Every other day for 5 days, AMB Media carriers were washed with sterile saline, TSB was aspirated, and the fresh broth was added. Biofilms formed on AMB Media carriers after 5 days were transferred to a 250 ml Erlenmeyer flask, which simulated an MBBR (Moving bed biofilm reactor). Each flask contained 200 ml of TSB medium (diluted 1:3) and 7 sterile AMB Media. HM solutions were added to the flasks so that each flask contained 200 μg/ml of the tested metal. The inoculation was carried out by adding one AMB Media with the formed biofilm to the Erlenmeyer flask. The flasks were incubated for 10 days at 26°C.

#### Quantification of the biofilm density

On the 1^st^, 5^th^, and 10^th^ days, the biofilm was quantified using the Bradford protein test (Kruger, 2002). A method for calculating the biomass of biofilm produced on an AMB media entails employing sonication to remove the biofilm from the carrier (an ultrasonic bath in the presence of phosphate buffer). The 2 ml of the sonicated sample was centrifuged at 10,000 rpm for 10 min. By adding 100 μl of physiological saline, the cell pellet was resuspended. The tubes were placed in a water bath and allowed to boil for 10 min in order to “dissolve” the proteins. Aliquots of the standards and samples (20 μl) were put into the tubes, and 1 ml of Bradford (biofilm reagent) was added. The absorbance was read on a UV–VIS spectrophotometer (OD_595_). Bovine serum albumin (BSA) was used to construct a standard curve with a concentration range of 125–1,250 μl/ml.

#### The removal and quantification of HMs ions from the medium

The removal of HMs ions using biofilm, formed on AMB Media carriers, was determined by a biosorption test in Erlenmeyer flasks that simulated MBBR. The experiment lasted 10 days. On the 1^st^, 5^th^, and 10^th^ days, 1 ml of sample from each flask was taken and centrifuged for 20 min at 10,000 rpm to remove microbial biomass and suspended particles. The supernatant of the sample was then filtered through a syringe filter (0.22 μm) and analyzed by an atomic absorption spectrophotometer (FAAS) to obtain the amount of metal remaining in the solution of the medium (Basak et al., 2014).

The flame atomic absorption spectrophotometer (FAAS) model Perkin Elmer 3,300 with a D_2_ lamp as a corrector was used for the determination of metals: Zn^2+^ (213.9 nm), Cu^2+^ (324.8 nm), Ni^2+^ (232.0 nm), Pb^2+^ (283.3 nm), and Cd^2+^ (228.8 nm). A calibration curve was prepared using standard solutions of suitable concentrations of HMs. The range of standard solutions was 0.5–2.0 mg/l for Cu^2+^, Zn^2+^, Cd^2+^, Ni^2+^ and 1.0–5.0 mg/l for Pb^2+^. All samples were analyzed by FAAS using an acetylene flame (2.0, 10.0). The measured values of the elements contained in the samples are expressed in mg/kg of dry matter.

At the specified conditions of detection, the detection limits for metals were: Zn^2+^ (0.5 ppm), Cu^2+^ (0.5 ppm), Ni^2+^ (1.0 ppm), Pb^2+^ (1.0 ppm), Cd^2+^ (0.5 ppm).

### Statistical analysis

All analyses were conducted using SPSS version software (IBM SPSS Statistics 22). For all experiments, statistical analysis was expressed as mean ± SD of triplicate (independent) experiments. The Wilcoxon’s test was used to checks whether the mean values of two dependent groups differ significantly from each other. *p* < 0.05 was considered a statistically significant difference.

## Results

### Microorganisms: Isolation, identification, and growth conditions

The isolated bacteria (Table 1) are facultative anaerobes, Gram negative, rod shaped, without pigments, and grow at 26 and 37°C. They ferment lactose and glucose and are oxidase negative and catalase positive. They grow on KCN and perform nitrate reduction. The Methyl Red test is negative and the Voges-Proskauer test is positive. Triple Sugar Iron Agar gives results: acid/acid; Gas positive; H_2_S negative. They use acetates, arabinose, cellobiose, maltose, raffinose, tartrate, trehalose, xylose, glycerol, salicin, and hydrolyzed esculin. Identification was made by the characteristics shown in Supplementary Table S1 and the confirmatory test (API 20E) is given in Supplementary Table S2 (with >96.5% probabilities).

**Table 1:** Single and mixed cultures used in the study.

Single microbial strains with isolation code	Mark	Mixed cultures	Mark
*Enterobacter cloacae* PMFKG-CV3	-	*E. cloacae/K. oxytoca*	EK
*Klebsiella oxytoca* PMFKG-CV4	-	*E. cloacae/S. odorifera*	ES
*Serratia odorifera* PMFKG-CV7	-	*E. cloacae/S. cerevisiae*	EC
*Saccharomyces cerevisiae* PMFKG-CV10	-	*K. oxytoca/S. odorifera*	KS
		*K. oxytoca/S. cerevisiae*	KC
		*E. cloacae/K. oxytoca/S.odorifera*	EKS
		*E. cloacae/K. oxytoca/S. cerevisiae*	EKC
		*K. oxytoca/S. odorifera/S. cerevisiae*	KSC
		*E. cloacae/S. odorifera/S. cerevisiae*	ESC

### Resistance of planktonic cells and their biofilms to HMs

Planktonic microorganisms show sensitivity to the presence of Cd^2+^ (MICs <15.62–250 μg/ml), while they show significant resistance to Pb^2+^, Cu^2+^, and Ni^2+^ (МICs 250 - >1,000 μg/ml) (Table 2). *K. oxytoca* shows the highest resistance to all tested metals, with the exception of Cd^2+^. The minimum biofilm eradication concentration (MBEC) for all microorganisms and for all tested metals was >1,000 μg/ml.

**Table 2:** Resistance of isolated and identified microorganisms in planktonic form to selected HMs expressed as MIC and MLC.

Microbial cultures	Test	Heavy metal (μg/mL)				
		Pb^2+^	Zn^2+^	Cd^2+^	Ni^2+^	Cu^2+^
*E. cloacae*	MIC^1^	125^Aa^	250^BCa^	250^CBa^	500^DEa^	500^EDa^
	MLC^2^	>1000^Db^	1000^ABCb^	>1000^Eb^	1000^BACb^	1000^CABb^
*K. oxytoca*	MIC	500^BCDEa^	31.25^Aa^	500^CBDEa^	500^DBCEa^	500^EBCDa^
	MLC	>1000^CDEb^	500^Ab^	>1000^DCEb^	1000^Bb^	>1000^ECDb^
*S. odorifera*	MIC	500^Ca^	250^ABa^	1000^Da^	250^BAa^	>1000^Eab^
	MLC	>1000^BDEb^	>1000^CBDEb^	>1000^DBCEb^	1000^Ab^	>1000^EBCDba^
*S. cerevisiae*	MIC	500^Ba^	125^Aa^	500^Ca^	500^Da^	>1000^Eab^
	MLC	>1000^CDEb^	1000^ABb^	>1000^DCEb^	1000^BAb^	>1000^ECDba^

Means denoted by a different capital letters in the same row (MIC and MLC) among treatments are significantly different (*p* < 0.05). Means denoted by a different small superscript letters in the same column (among MIC and MLC) for each biofilm are significantly different (*p* < 0.05).

1 МIC, minimum inhibitory concentration.

2 МLC, minimal lethal concentration.

### Assay for microbial interaction and biofilm formation

After isolation, a total of four single microbial strains and nine mixed cultures (defined in Table 1) were allowed to form biofilms in control growth medium.

**Figure 1:**
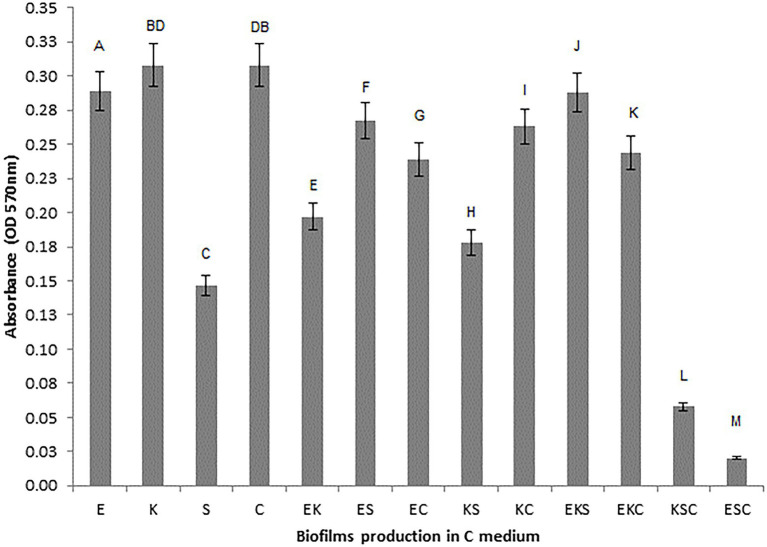
Growth control of the single-and the mixed- biofilms in polystyrene microtiter plates: (E) *E. cloacae*, (K) *K. oxytoca*, (S) *S. odorifera*, (C) *S. cerevisiae*, (EK) *E. cloacae/K. oxytoca*, (ES) *E. cloacae/S. odorifera*, (EC) *E. cloacae/S. cerevisiae*, (KS) *K. oxytoca/S. odorifera*, (KC) *K. oxytoca/S. cerevisiae*, (EKS) *E. cloacae/K. oxytoca/S.odorifera*, (EKC) *E. cloacae/K. oxytoca/S. cerevisiae*, (KSC) *K. oxytoca/S. odorifera/S. cerevisiae*, (ESC) *E. cloacae/S. odorifera/S. cerevisiae*. All results are expressed as the mean ± SE. The statistical test was based on Wilcoxon’s test. Different capital letters above means indicate significant differences among biofilms at a threshold of *p* < 0.05.

To investigate the microbial interaction patterns between strains, the biomasses of single-and mixed-species biofilms were measured in the growth liquid medium. The growth control of the biofilms in polystyrene microtiter plates is shown in Figure 1. In control growth conditions, the best biofilm producing strains were *K. oxytoca* and *S. cerevisiae* (0.308), followed by *E. cloacae* (0.289) and *S. odorifera* (0.147), whereas the mixed cultures showed a weaker ability to form a biofilm than single strains, except *S. cerevisiae*. Among mixed cultures, three-species culture EKS produced the highest biomass (0.288), followed by two co-culture species, ES (0.267) and KS (0.263). The next range of biomass productivity was achieved by EKC (0.244), EC (0.239), EK (0.197), and KS (0.178). Finally, very low biomass productivity was observed in medium inoculated with three co-culture species: KSC and ESC. Compared to single strains, the biofilm biomass of mixed cultures was slightly or noticeably lesser or higher depending on the microbial strain. For example, in the case when *S. odorifera* was co-cultured with *E. cloacae* and *K. oxytoca*, the mixed biofilm biomass was notable higher than the biomass of a single *S. odorifera* and noticeably lesser than the single *E. cloacae* and *K. oxytoca*. The biomass of the EKS mixed culture was plentiful higher than the biomass of *S. odorifera*, but almost identical to the biomass of *E. cloacae* and slightly lesser than *K. oxytoca.* However, the mixed biofilms ESC and KSC showed the lowest biofilm production, whereas the single species that built them showed significantly greater potential in biofilm formation.

### The determination level of HMs resistance of tested biofilms

The level of microbial biofilm resistance in the presence of HMs was determined and expressed through minimum biofilm inhibitory concentration (MBIC) and minimum biofilm eradication concentration (MBEC). From the results shown in Table 3, it is apparent that among single strains, the highest resistance was expressed by *E. cloacae*, with MBEC ≥100,000 μg/ml toward Pb^2+^, Zn^2+^, Cd^2+^, and Ni^2+^, followed by *K. oxytoca* and *S. odorifera*, which were resistant to Zn^2+^, Cd^2+^, and Ni^2+^. Among co-cultures, the best resistance ability was achieved by KC (toward Pb^2+^, Zn^2+^, Cu^2+^, Ni^2+^), followed by ES (toward Pb^2+^, Zn^2+^, Ni^2+^), EK, and EC (toward Zn^2+^, Ni^2+^). Mixed cultures composed of 3 species showed the highest resistance to the presence of all tested metals, excluding ESC (MBIC<781 μg/ml).

**Table 3:** Degree of resistance for single-and mixed biofilms in the presence of selected HMs.

Microbial cultures	Test	Heavy metal (μg/mL)				
		Pb^2+^	Zn^2+^	Cd^2+^	Ni^2+^	Cu^2+^
*E. cloacae*	MBIC^1^	781^Aa^	12500^Ca^	25000^Da^	6250^Ba^	50000^Ea^
	MBEC^2^	100000^BCDEb^	100000^CBDEb^	50000^Ab^	100000^DBCEb^	100000^EBCDb^
*K. oxytoca*	MBIC	781^Aa^	6250^Ca^	25000^DEab^	1562^Ba^	25000^EDa^
	MBEC	25000^Ab^	100000^CEb^	25000^Bba^	>100000^DCEb^	100000^ECDb^
*S. odorifera*	MBIC	1562^Aa^	25000^EDa^	25000^DEab^	6250^Ca^	3125^Ba^
	MBEC	50000^BCb^	100000^DEb^	25000^Aba^	100000^EDb^	50000^CBb^
*S. cerevisiae*	MBIC	781^Aa^	25000^DEa^	12500^Cab^	25000^EDa^	1562^Ba^
	MBEC	25000^Bb^	100000^CEb^	12500^Aba^	>100000^DCEb^	100000^ECb^
EK	MBIC	1562^Aa^	12500^CDEa^	3125^Ba^	12500^DCEa^	12500^ECDa^
	MBEC	50000^Cb^	100000^Db^	3125^Aba^	>100000^Eb^	25000^Bb^
ES	MBIC	781^Aa^	12500^DEa^	6250^Bab^	6250^CBa^	12500^EDa^
	MBEC	100000^CDb^	100000^DCb^	6250^Aba^	>100000^Eb^	50000^Bb^
EC	MBIC	1562^Aa^	12500^CDa^	25000^Eab^	12500^DCa^	6250^Ba^
	MBEC	25000^ABb^	100000^Db^	25000^BAba^	>100000^Eb^	50000^Cb^
KS	MBIC	1562^Aa^	6250^Ba^	12500^CDa^	12500^DCa^	50000^Ea^
	MBEC	50000^BCb^	100000^Db^	50000^CBb^	>100000^Eb^	12500^Ab^
KC	MBIC	3125^ABa^	6250^CDa^	50000^Eab^	6250^DCa^	3125^BAa^
	MBEC	>100000^EDb^	100000^BCb^	50000^Aba^	>100000^DEb^	100000^CBb^
EKS	MBIC	1562^ABa^	1562^BAa^	>100000^Eab^	50000^Da^	6250^Ca^
	MBEC	100000^BCb^	100000^CBb^	>100000^DEba^	>100000^EDb^	50000^Ab^
EKC	MBIC	781^Aa^	6250^Ba^	>100000^Eab^	25000^Da^	12500^Ca^
	MBEC	100000^BCba^	100000^CBb^	>100000^DEba^	>100000^EDb^	25000^Ab^
KSC	MBIC	12500^Aa^	50000^Ba^	>100000^DEab^	>100000^EDab^	100000^Ca^
	MBEC	100000^BCb^	100000^CBb^	>100000^DEba^	>100000^EDba^	50000^Ab^
ESC	MBIC	<781^BCDEab^	<781^CBDEab^	<781^DCBEab^	<781^EDBCab^	781^Aa^
	MBEC	<781^ABCDba^	<781^BACDba^	<781^CABDba^	<781^DABCba^	1562^Eb^

The measurements are made in duplicate. The values are constant. Means denoted by a different capital letters in the same row (MBIC and MBEC) among treatments are significantly different (*p* < 0.05). Means denoted by a different small superscript letters in the same column (among MBIC and MBEC) for each biofilm are significantly different (*p* < 0.05).

1 MBIC, minimum biofilm inhibitory concentration.

2 MBEC, minimum biofilm eradication concentration.

Generally, the tested biofilms showed high levels of resistance to Zn^2+^ and Ni^2+^, except the biofilm of ESC. Toward Cu^2+^, the high levels of resistance were shown all single strains with the exception of *S. odorifera* and co-culture KC. Mixed communities of three species, including EKS, EKC, KSC and two co-cultures, ES and KC, were the most resistant to Pb^2+^. Single-and co-cultures were both extremely sensitive to Cd^2+^, whereas three-species cultures were extremely resistant (with the exception of ESC). It is worth noting that the mixed biofilm ESC showed extremely high sensitivity (MBEC = 781 and 1,562 μg/ml) to the tested HMs. Some biofilms’ sensitivity was HM type specific, so EK and ES were sensitive to Cd^2+^ at MBEC = 3,125 and 6,250 μg/ml, respectively. Also, KS was most sensitive toward Cu^2+^ (MBEC = 12,500 μg/ml), whereas *K. oxytoca, S. odorifera* and EC were most sensitive toward Pb^2+^ (MBEC = 25,000 μg/ml).

### Single-and mixed-biofilms formation on AMB media carriers in control and HMs polluted media

**Figure 2:**
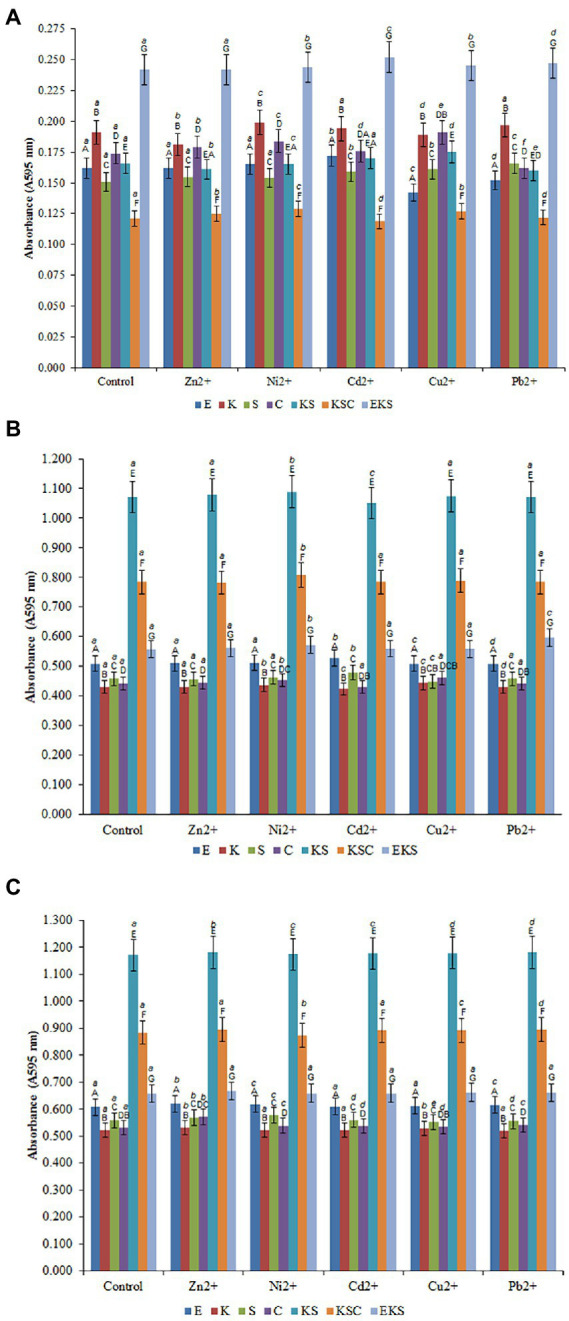
Development of the biofilms on AMB Media carriers in untreated growth control and in the presence of heavy metals after 1st **(A)**, 5th **(B)** and 10th **(C)** days. (E) *E. cloacae*, (K) *K. oxytoca*, (S) *S. odorifera*, (C) *S. cerevisiae*, (EK) *E. cloacae/K. oxytoca*, (ES) *E. cloacae/S. odorifera*, (EC) *E. cloacae/S. cerevisiae*, (KS) *K. oxytoca/S. odorifera*, (KC) *K. oxytoca/S. cerevisiae*, (EKS) *E. cloacae/K. oxytoca/S.odorifera*, (EKC) *E. cloacae/K. oxytoca/S. cerevisiae*, (KSC) *K. oxytoca/S. odorifera/S. cerevisiae,* (ESC) *E. cloacae/S. odorifera/S. cerevisiae*. All results are expressed as the mean ± SE. The statistical test was based on Wilcoxon’s test. Different capital letters above means indicate significant differences among biofilms inside treatments at a threshold of *p* < 0.05. Different small letters above means of biofilms among treatments are significantly different (*p* < 0.05).

The biofilms that exhibited the highest resistance in the presence of tested HMs were selected to monitor biosorption efficiency. The biofilms of *E. cloacae*, *K. oxytoca*, *S. odorifera*, *S. cerevisiae*, KS, KSC and EKS were selected for further testing. The results of the biofilms growth on AMB Media carriers in the presence of HMs were investigated, compared to the control (without metals) and presented in Figures 2A–C.

Figures 2A–C depict the formation of individual and mixed culture biofilms after the 1st, 5th, and 10th days after inoculation in the control medium and the media with the addition of HMs at a concentration of 200 μg/ml. The formation of biofilms after the 1st day of inoculation in the control media may be depicted as follows: EKS (0.242) > *K. oxytoca* (0.191) > *S. cerevisiae* (0.174) > KS (0.166) > *E. cloacae* (0.162) > *S. odorifera* (0.151) > KSC (0.121) (Figure 2A). Compared to the control, the addition of Ni^2+^ ions stimulated the production of biofilm *S. cerevisiae*, whereas Cd^2+^ ions stimulated the production of biofilm *E. cloacae*. Pb^2+^ stimulated the production of biofilm *S. cerevisiae* whereas Cu^2+^ had a stimulating effect on biofilms *S. odorifera*, *S. cerevisiae*, and KSC. Conversely, the addition of Zn^2+^, Cu^2+^, and Pb^2+^ ions inhibited the production of biofilms *K. oxytoca*, *E. cloacae*, and *E. cloacae* and *S. cerevisiae*, respectively (Figure 2A).

All examined cultures showed a significant increase in biofilm biomass after the 5th day of incubation in the control media. As Figure 2B shows, KS generated the highest biofilm biomass (1.072), followed by KSC (0.784), EKS (0.557), *E. cloacae* (0.508), *S. odorifera* (0.458), *S. cerevisiae* (0.441) and *K. oxytoca* (0.430). The addition of Ni^2+^ to the medium stimulated the production of all biofilms, particularly KSC and KS. Cd^2+^ ions stimulated the growth of biofilms *E. cloacae* and *S. odorifera*, but inhibited the growth of biofilms *K. oxytoca*, *S. cerevisiae*, and KS. The ions of Cu^2+^ had a stimulating effect on the growth of all biofilms except *S. odorifera*, whereas Pb^2+^ stimulated only the EKS biofilm (Figure 2B).

After the 10th day of incubation, the production of biofilms of all tested cultures was higher compared to the previous cultivation period (Figure 2C). The highest biofilm productivity was achieved by KS (1.172), followed by KSC (0.884), EKS (0.657), *E. cloacae* (0.608), *S. odorifera* (0.558), *S. cerevisiae* (0.531) and *K. oxytoca* (0.521). The presence of tested HMs in the medium stimulated the growth of biofilm biomass of microbial cultures with some exceptions, such as Ni^2+^ ions inhibited the growth of biofilm KSC, while ions Cu^2+^ and Pb^2+^ inhibited the growth of biofilm *S. odorifera*.

### Biosorption assay of single and mixed biofilms

Biosorption of HMs, namely Pb^2+^, Zn^2+^, Cd^2+^, Cu^2+^, and Ni^2+^, was conducted using both individual and mixed biofilms. The results of HMs removal are shown in Table 4. On the 1st day of the biofilm incubation, HMs were added separately at a concentration of 200 μg/ml, followed by the concentration of HMs was monitored after the 5th and 10th days of incubation. By measuring the amount of HMs remaining in the substrate on the 1st day, no changes in the amount of HMs were detected in the substrate. However, after 5 days, the amount of HMs was changed compared to the 1st day of incubation. The amount of HMs ions measured on the 5th day ranged from 6.71 to 11.27 μg/ml for Cd^2+^, from 4.25 to 6.41 μg/ml for Ni^2+^, from 3.88 to 26.01 μg/ml for Pb^2+^, from 1.92 to 5.51 μg/ml for Cu^2+^, and from 4.22 to 11.76 μg/ml for Zn^2+^. By transforming these values into percentages, in media with the addition of Cd^2+^, biosorption values ranged from 94.37 to 96.65% depending on biofilm species. At the same time, the measured biosorption values of Ni^2+^ were 96.79–97.88%, whereas the biosorption values of Pb^2+^, Cu^2+^ and Zn^2+^ were 86.99–98.06, 97.25–99.04, and 93.54–97.89%, respectively. Based on the results presented in Table 4, the most effective biosorbents were KSC and EKS, followed by EKS, and KSC for Cu^2+^, Pb^2+^, Zn^2+^, Ni^2+^ and Cd^2+^, respectively. After 10 days, the quantity of HMs ions in the media varied 9.76–1.94 μg/ml for Cd^2+^, 5.28–3.55 μg/ml for Ni^2+^, 13.69–1.73 μg/ml for Pb^2+^, 4.23–1.63 μg/ml for Cu^2+^, and 12.83–3.88 μg/ml for Zn^2+^. Obviously, the biosorption capacity of biofilms for HMs insignificantly increased after 10 days in the following manner: Cd^2+^ values ranged from 96.02–99.03; Ni^2+^ values ranged from 97.36–98.47%; Pb^2+^ values ranged from 93.16–99.13%; Cu^2+^ values ranged from 97.89–99.19%, and Zn^2+^ values ranged from 93.58–98.06% (Table 4). The best biosorption capacity was shown by KSC for Cu^2+^, followed by EKS for Pb^2+^, and Cd^2+^, *K. oxytoca* for Ni^2+^, and *E. cloacae* for Zn^2+^.

**Table 4:** Biosorption potential of the single-and mixed biofilms for removal tested HMs.

Biofilm	Days	μg/mL					%				
		Cd^2+^	Ni^2+^	Pb^2+^	Cu^2+^	Zn^2+^	Cd^2+^	Ni^2+^	Pb^2+^	Cu^2+^	Zn^2+^
*E. cloacae*	1	200^C^	200^C^	200^C^	200^C^	200^C^	0	0	0	0	0
	5	9.99^Be^	4.95^ABcb^	9.49^Bd^	4.86^Bbc^	4.22^Ba^	95.01	97.53	95.26	97.57	97.89
	10	4.38^Ade^	4.48^ABed^	2.10^Aa^	3.94^Acb^	3.88^Abc^	97.81	97.76	98.95	98.03	98.06
*K. oxytoca*	1	200^C^	200^C^	200^C^	200^C^	200^C^	0	0	0	0	0
	5	11.27^Bd^	4.36^Aba^	10.38^Bc^	4.32^Bab^	12.91^BAe^	94.37	97.82	94.81	97.84	93.54
	10	7.96^Ad^	5.06^Bb^	7.21^Ac^	3.09^Aa^	12.83^Ae^	96.02	98.47	96.39	98.00	93.58
*S. odorifera*	1	200^C^	200^C^	200^C^	200^C^	200^C^	0	0	0	0	0
	5	10.77^Bd^	6.41^Bb^	26.01^Be^	4.03^Ba^	9.32^Bc^	94.61	96.79	86.99	97.98	95.34
	10	6.07^Adc^	5.28^Ab^	13.69^Ae^	3.55^Aa^	5.91^Ac^	96.96	97.36	93.16	98.22	97.04
*S. cerevisiae*	1	200^C^	200^C^	200^C^	200^C^	200^C^	0	0	0	0	0
	5	10.35^Bed^	6.21^Bcb^	6.21^Bbc^	5.51^Ba^	10.33^Bde^	94.82	96.90	96.89	97.25	94.84
	10	9.76^Ae^	3.55^Ab^	2.37^Aa^	4.23^Ac^	7.31^Ad^	95.12	98.23	98.82	97.89	96.34
KS	1	200^C^	200^C^	200^C^	200^C^	200^C^	0	0	0	0	0
	5	7.26^Bc^	4.25^BAb^	11.47^Be^	3.69^BAa^	8.90^Bd^	96.37	97.88	94.27	98.15	95.55
	10	2.99^Ab^	3.98^Ac^	4.48^Ad^	2.37^Aa^	8.14^Ae^	98.51	98.01	97.76	98.82	95.93
KSC	1	200^C^	200^C^	200^C^	200^C^	200^C^	0	0	0	0	0
	5	6.71^Bc^	4.74^Bb^	15.21^Be^	1.92^BAa^	11.76^Bd^	96.65	97.63	93.40	99.04	94.12
	10	2.06^Ab^	3.66^Ac^	9.69^Ae^	1.63^Aa^	4.67^Ad^	98.97	98.17	95.15	99.19	97.66
EKS	1	200^C^	200^C^	200^C^	200^C^	200^C^	0	0	0	0	0
	5	7.35^Bd^	4.25^BAcb^	3.88^Bbac^	3.57^Bba^	11.73^Be^	96.32	97.88	98.06	98.22	94.14
	10	1.94^Aba^	4.08^Ad^	1.73^Aab^	2.48^Ac^	8.02^Ae^	99.03	97.96	99.13	98.76	95.99

The measurements are made in duplicate. The values are constant. Means in the same column of each biofilm among days marked with capital letters in superscript are significantly different (*p* < 0.05). Means in the same row of each biofilm between treatments marked with small letters in superscript are significantly different (*p* < 0.05).

When the efficiency of biofilms in the biosorption of HMs was compared, EKS was the most efficient Cd^2+^ biosorbent, whereas other biofilms were less efficient: *E. cloacae* and *S. cerevisiae* approximately 1.2 and 1.3 fold, KS 2.6 fold, *K. oxytoca* 4.2 fold, KSC 5.8 fold, and *S. odorifera* approximately 8 fold. Biofilm *K. oxytoca* removed the most Ni^2+^, while biofilm *S. cerevisiae* and *KSC* were less effective (about 1.2 fold). Biofilms KS, EKS, *E. cloacae*, and *S. odorifera* demonstrated reduced efficiency ranging from 1.3 to 1.7 fold in the same assay. Biofilm EKS demonstrated the greatest biosorption property for Pb^2+^, but the other biofilms, namely *E. cloacae*, *S. cerevisiae*, KS, KSC, and *S. odorifera*, were weaker (about 1.4, 2.6, 4, 5.6, and 8 fold, respectively). The KSC was the most efficient in Cu^2+^ biosorption, whereas EKS and *S. odorifera* were approximately 1.5 fold weaker and *E. cloacae*, *K. oxytoca*, and *S. cerevisiae* were 2 to 2.6 fold weaker. The most effective Zn^2+^ biosorbent was *E. cloacae*, whereas KSC and *S. cerevisiae* were 1.2–1.5 fold weaker, *S. cerevisiae*, EKS and KS were 2 fold, and *K. oxytoca* was 3.3 fold weaker.

## Discussion

### Microbial interaction and biofilm formation

During the last decade, the mutual interaction of microorganisms and their ability to form biofilms in different nutrient media were the subject of numerous studies. The results of these studies performed on the biofilm formation of single and mixed-species communities are numerous and contradictory. Some researchers confirmed the best biofilm production by individual strains compared to their mixed cultures due to their antagonistic relationship. In contrast, numerous studies have established that synergism among different microbial strains leads to enhanced growth of mixed cultures compared to single cultures (Ren et al., 2015; Berlanga and Guerrero, 2016). This is consistent with the concept that self-organization and cooperation, rather than competition among individual microorganisms, are crucial for bacterial communities to survive (Luo et al., 2022). Regarding these statements, the current study investigated and compared the production of biofilms by single strains and their combinations in control liquid medium. Such results showed the best biofilm production by single strains compared to mixed communities, which is the opposite of the previously mentioned statement. The strains identified as the best biofilm producers are *K. oxytoca* and *S. cerevisiae*, while *E. cloacae* and *S. odorifera* produced less biomass (6.17 and 52.27%, respectively). Among two-species cultures, ES and KC achieved the highest and almost identical biomass, whereas EC, EK, and KS produced noticeably lower biomass (11.49, 25.38, and 33.33%, respectively). From three-species mixed cultures, EKS and EKC produced a significant amount of biomass in such a way that EKC biomass was lesser (15.28%) compared to EKS. Mixed communities marked as KSC and ESC produced a very small quantity of biofilms, which indicates strong competition among single strains of communities. Competitive or antagonistic relationships among species within the biofilm are the result of similar metabolic pathways and nutritional resources (Elias and Banin, 2012) or the production of some inhibitory compounds. The study by Hoffman et al. (2006) found that competitive conditions in the biofilm reflected inhibition of development of EPS and biofilm biomass to a certain degree.

### The determination level of HMs resistance of tested biofilms

Investigations of the effects of the HMs on bacterial biofilms confirmed the role of the EPS matrix in biofilm resistance. Some authors (Hullebusch et al., 2003; Harrison et al., 2007) revealed the negatively charged EPS matrix attaches positively charged metal ions and acts as a diffusion barrier, limiting or at least delaying ion penetration into the biofilm. Geesey and Jang (1989) found that the effect of HM ions on cells depends on their concentration since the fluctuations within a millimolar range between growth promoting and toxic effects were detected. In this study, HMs susceptibility was assayed in two ways: inhibition of growth (MBIC) and lethality (MBEC). The MBICs of the tested HMs were determined for all biofilms. Among all biofilms, only biofilm ESC had a MBIC value less than 781 μg/ml for all tested HMs, except Cu^2+^. A MBIC value of 781 μg/ml was found for Pb^2+^ (*E. cloacae*, *K. oxytoca*, *S. cerevisiae*, ES, KSC) and Cu^2+^ (ESC), 1,562 μg/ml for Zn^2+^ (EKS), Ni^2+^ (*K. oxytoca*) and Cu^2+^ (*S. cerevisiae*), 3,125 μg/ml for Cd^2+^ (EK). A MBEC values higher of 100,000 μg/ml were determined for Pb^2+^ (KS), Zn^2+^ (EKS, EKC, KSC), Ni^2+^ (*K. oxytoca*, *S. cerevisiae*, EK, ES, EC, KS, KC, EKS, EKC, KSC). The HMs resistance results were in agreement with a study investigating the tolerance of natural, mixed biofilm in the presence of HMs from tailings ponds (Pb^2+^, Ni^2+^, Cu^2+^, etc.). Golby et al. (2014) found that *Rhodococcus erythropolis* isolated from the community was less tolerant in the presence of metals compared to mixed biofilm, so it was unable to perform biomineralization of metals. By comparing the individual and mixed biofilms of *Rhodotorula mucilaginosa* and *Escherichia coli,* Grujić et al. (2017b) found a higher resistance of mixed biofilms to the presence of HMs. Scientists confirmed that different regulatory processes (biochemical and genetic) take place within the biofilm that allow microorganisms to develop a better mechanism of tolerance and improve their resistance in the presence of HMs (Harrison et al., 2007). Hullebusch et al. (2003) described that EPS components offer a high concentration of charged functional groups, such as hydroxyl-, phosphoryl-, carboxyl-, and amino groups, which have the ability to bind and immobilize metal ions. As observed in hydrogen-producing sludge and sulfate-reducing biofilms, the number of electrostatic binding sites in the EPS matrix is 20-to-30-fold higher than on the bacterial surface and this reduces or even prohibits metal permeability (Liu and Fang, 2002a,b).

### Single-and mixed-biofilms formation on AMB media carriers in control and HMs polluted media

The influence of different surfaces on the formation of biofilms of microbial communities isolated from wastewater was investigated for various types of bacteria, mainly from the genera *Bacillus* and *Pseudomonas* (Meyer and Wallis, 1997). One of the main conclusions was that the biofilm develops well on granular (ground) glass and polystyrene strips, while on smooth glass the biofilm does not form, or occurs in a very small number of cells, which indicates that polystyrene tapes would be a practical and economical system for the removal of HMs in large quantities. The findings of the aforementioned investigation served as the foundation for the current study’s selection of materials for biofilm development and assessment of both the tolerance and biosorption of the selected HMs. Based on obtained results, all selected microorganisms have demonstrated the ability to form biofilms on AMB Media carriers both individually and as co-cultures composed of two or three species. The concentration of HMs did not interfere with the growth and production of single and mixed biofilms. The growth of single and mixed biofilms after the 1st day in the control medium was similar to the production of biofilms in the HMs polluted media. The highest growth was observed for EKS biofilm, both in the control and metal-polluted media, regardless of the metal type. A slight growth effect was demonstrated after the 1st day in medium with Cu^2+^ on the *E. cloacae* biofilm. The differences that were observed after the 5th day concern the production of biofilms between the species of microorganisms that build them. Up to the 10th day, biofilms continued to grow both in the control and HM polluted media, but at a reduced rate. Among the individual species, the most significant growth was recorded by the biofilm *E. cloacae*. Literature data about microbial biofilm growth in the presence of HMs are mainly focused on single strains. Haque et al. (2021) found that the amount of biofilm production by *Enterobacter asburiae* ENSD102, *Enterobacter ludwigii* ENSH201, *Vitreoscilla* sp. ENSG301, *Acinetobacter lwoffii* ENSG302, and *Bacillus thuringiensis* ENSW401 were 11.5, 11.6, 11.6, 11.6, and 11.7 mg/ml, respectively, in response to 200 mg/l Cu^2+^. In the case of Ni^2+^, it varied from 11.7, 11.5, 11.7, 11.6, and 11.8 mg/ml in *E. asburiae* ENSD102, *E. ludwigii* ENSH201, *Vitreoscilla sp*. ENSG301, *A. lwoffii* ENSG302, and *B. thuringiensis* ENSW401, respectively. *E. asburiae* ENSD102, *E. ludwigii* ENSH201, *Vitreoscilla sp.* ENSG301, *A. lwoffii* ENSG302, and *B. thuringiensis* ENSW401 produced biomass biofilms in 200 mg/l Pb^2+^ at 9.7, 9.9, 9.5, and 9.7 mg/ml, respectively. Syed et al. (2021) concluded that *E. cloacae* MC9’s biofilm-forming capacity was substantially decreased at 200 μg/ml concentration of Cd^2+^, Cr^2+^, Pb^2+^, and Ni^2+^ by 73, 64, 51, and 42%, respectively, compared to control. In this study, the resistance/sensitivity of the biofilm of *E. cloacae* to Cu^2+^ and Zn^2+^ as well as the biosorption effect were tested for the first time. The current results confirmed the excellent capacity of *E. cloacae* biofilm to remove tested HMs from the medium. In addition, by mixing *E. cloacae, K. oxytoca,* and *S. odorifera,* resulted in the production of a biofilm highly resistant to the tested metals and a power potential for reducing HMs in waste water.

### Biosorption efficiency of single and mixed biofilms

The effects of different parameters on the biosorption ability of microbial biofilm to the presence of various HMs are well studied and documented in the literature. Wang and Chen (2006) discovered that *S. cerevisiae* can remove Pb^2+^ and uranium more efficiently compared to other metals. For this yeast, the biosorption capacity was highly dependent on various parameters: pH, the ratio of initial metal concentrations and biomass, the presence of the different metal ions in solution, and, in some cases, temperature. Ghorbani et al. (2008) investigated the optimization of the Cd^2+^ biosorption process by varying three parameters: pH, ion concentration, and *S. cerevisiae* amount, concluding that one-layer adsorption mechanisms with diffusion within the particles were effective in the Cd^2+^ biosorption process. The current study showed that *S. cerevisiae* had the highest biosorption rate in the presence of Pb^2+^, Cu^2+^, and Ni^2+^, which is in line with the previous studies by other authors (Ozer and Ozer, 2003). The obtained results are much better than the results published in the study by Rahatgaonkar and Mahore (2008). The mentioned authors explained that *S. cerevisiae* biomass was reduced by Cd^2+^, Ni^2+^, Cu^2+^, and Co^2+^, up to 63, 50, 44, and 80%, respectively. Chen and Wang (2007) used *S cerevisiae* as waste biomass for the adsorption of many types of HMs, and obtained the maximum biosorption capacity in the following order: Pb^2+^ > Cu^2+^ > Zn^2+^ > Cd^2+^ > Ni^2+^. The current result partially matches with the mentioned research because the biosorption potential of the isolated yeast has the following capacity: Pb^2+^ > Ni^2+^ > Cu^2+^ > Zn^2+^ > Cd^2+^. Obviously, Ni^2+^ ions are absorbed better by yeast than Cu^2+^ ions. The obtained results could be explained by the fact that the yeast biofilm in the current study was a live system, in contrast to the biomass used in the previous study. Apart from this, the adoption of the other metals (Chen and Wang, 2007) is in accordance with our results. Heavy metal ion removal by yeast can occur both on the cell surface and within live cells. The mechanism is complicated, involving both passive and active adsorption (He et al., 2007). *S. cerevisiae* identified as a viable candidate for Zn^2+^ wastewater treatment. The major processes of metal removal from solution are surface adsorption, chemisorption, and ion exchange (Zinicovscaia et al., 2020).

Differences in biosorption of HMs could be caused by differences in chemical structure as well as the characteristics of the microorganism, such as the structure of the cell wall, functional groups, and surfaces. Bacterial cell walls are mostly made of peptidoglycans, a linear chain of the disaccharide N-acetylglucosamine-β1,4-Nacetylmuramic acid linked by peptide chains. Bacterial cell walls include functional groups such as carboxyl, phosphonate, amine, and hydroxyl groups that are responsible for binding actions (Vijayaraghavan and Yun, 2008). Holan and Volesky (1994) stated that sorption of the metals increases with increasing valence and the atomic number of metals, which was partially agreed upon by the current study. Orji et al. (2021) investigated the biosorption efficiency of *Klebsiella* sp. USL25 for removing Hg^2+^, Pb^2+^, Cd^2+^, and Ni^2+^ and obtained the following results: 85, 97.13, 73.33, and 86.06%, respectively. Khan et al. (2015) found a 40.15% removal ability of Cd^2+^ by *K. pneumonia.* The current results obtained for *K. oxytoca* are much better for Cd^2+^ and Ni^2+^, and very similar for Pb^2+^.

Moreover, the current study demonstrates that the HMs uptake from solution, as well as resistance in the presence of metals, were increased by mixed biofilms, with exceptions such as *E. cloacae* for Zn^2+^ and Pb^2+^; *S. cerevisiae* for Pb^2+^; and *K. oxytoca* for Ni^2+^. These are in accordance with the conclusion that microorganisms can be strictly specific in respect of only one or more metals. Grujić et al. (2017b) studied the metal removal efficiency for the *R. mucilaginosa* and *E. coli* and observed that the efficiency of the single biofilm ranged from 81.56–97.85%, compared to their mixed biofilms (94.99–99.88%). Golby et al. (2014) came to the same conclusion and indicate that mixed biofilms present a more practical tool in the remediation of contaminated environments than single ones.

The effect of incubation time on the biosorption capacity of various microorganisms is reported in the literature (Harrison et al., 2005a,b,c). According to Grujić et al. (2017b), *R. mucilaginosa* biofilm had maximal removal capacity for Cd^2+^, Ni^2+^, and Zn^2+^ (over 90%) during 48 h. Basak et al. (2014) recorded that 88 and 72.2% of Zn^2+^ were removed by *Candida rugosa* and *Cryptococcus laurentii* biofilms, respectively, in 24 h. The current study showed a significant difference in Cd^2+^ and Pb^2+^ removal efficiency between the 5th and 10th days of the experiment compared to the other HMs tested. In these two metals, adsorption took place even after 5 days with a significant proportion and was more effective by mixed than single biofilms. Finally, the current study established the excellent potential of single and mixed biofilms in the removal of HMs in the following manner: Cu^2+^ (KSC–99.19%) > Pb^2+^ (EKS–99.13%) > Cd^2+^ (EKS–99.03%) > Ni^2+^ (*K. oxytoca*–98.47%) > Zn^2+^ (*E. cloacae*–98.06%). Azizi et al. (2016) found that Cu^2+^ and Zn^2+^ are more readily removed than Cd^2+^ and Ni^2+^ (Cu^2+^ > Zn^2+^ > Ni^2+^ > Cd^2+^) using a PBBR biological system. With the exception of Cu^2+^, the results of this study are the opposite of previously reported results in terms of biosorption efficiency for reducing the tested metals. Uptake of metal ions is a complex mechanism of EPS release. EPS has a key role in stopping the penetration of metals into the intracellular environment where ion exchange can occur (Pham et al., 2022).

In addition to biosorption, living cells also use bioaccumulation to remove HMs. HMs are accumulated and taken into intracellular live bacterial cells using proteins. There they are converted into a non-bioavailable form by binding to metallothioneins. Such active adsorption is the intracellular accumulation of toxicants in living cells within the cytoplasm that depends on metabolism. Associated with HM, these intracellular proteins can also lower the concentration of free ions in the cytoplasm where metal detoxification has occurred (Etesami, 2018). A study by Ghosh et al. (2022) reported that *Enterobacter cloacae* can remove HMs through a bioaccumulation process. The efficiency of HMs accumulation depends not only on the internal structure of the cell and space, genetic characteristics, and cellular processes for enzymatic catalysis, but also on the location from which the bacterial strain is isolated, in natural areas with extreme conditions or grows with adaptation in a contaminated place (Pham et al., 2022). Microorganisms capable of accumulating HM can tolerate one or more metals at higher concentrations (Mosa et al., 2016). Therefore, this research will continue in the direction of molecular assessment of the most successful microorganisms as well as the discovery of positive interactions in the most successful combinations and mechanisms responsible for resistance and biosorption.

## Conclusion

The current study determined which autochthonous microorganism strains or their combinations are the most successful in the biosorption of HMs from the environment. The four individual and nine mixed cultures grown on the AMB carrier were transferred in medium with HMs: Cu^2+^, Zn^2+^, Cd^2+^, Ni^2+^, and Pb^2+^. After 10 days of incubation, KSC showed the maximum biosorption capacity for Cu^2+^ (99.19%), followed by EKS for Pb^2+^ (99.13%) and Cd^2+^ (99.03%), *K. oxytoca* for Ni^2+^ (98.47%), and *E. cloacae* for Zn^2+^ (98.06%). The results of this study offer new knowledge about the bisorption potential of autochthonous strain *E. cloacae* for Cu^2+^ and Zn^2+^ and highlight its usefulness in metal removal procedures. Furthermore, the discovery of the “ideal” combination of microorganisms with high biosorption effectiveness, such as KSC and EKS, is of great importance for the improvement of the bioremediation process and the elimination of HMs from the environment.

## Data availability statement

The original contributions presented in the study are included in the article/Supplementary material, further inquiries can be directed to the corresponding author.

## Author contributions

IR and VJ conceived and designed the experiments, analyzed the experimental data, and wrote the manuscript. SG collected waste water sample and conducted all the experiments in microbiology laboratory. ZS detected the amount of heavy metals by an atomic absorption spectrophotometer. AO participated in this work as consultants in process of manuscript revision. All authors contributed to the article and approved the submitted version.

## Funding

This work was supported by the Ministry of Education, Science and Technological Development of the Republic of Serbia (agreement nos. 451-03-68/2022-14/200122 and 451-03-9/2022-14/200378), and Multilateral Scientific and Technological Cooperation in the Danube region for 2020–2022 year (DS10) 337-00-00322/2019-09/107 metal microorganism′s interaction as a basic for progressive biotechnological processes.

## Conflict of interest

The authors declare that the research was conducted in the absence of any commercial or financial relationships that could be construed as a potential conflict of interest.

## Publisher’s note

All claims expressed in this article are solely those of the authors and do not necessarily represent those of their affiliated organizations, or those of the publisher, the editors and the reviewers. Any product that may be evaluated in this article, or claim that may be made by its manufacturer, is not guaranteed or endorsed by the publisher.
