# Supplementary material for: Finding the best combination of autochthonous microorganisms with the most effective biosorption ability for heavy metals removal from wastewater
Source: Front Microbiol. 2022 Oct 6;13:1017372. doi: 10.3389/fmicb.2022.1017372 (PMC9577556; doi:10.3389/fmicb.2022.1017372)
Supplement: Supplementary file 1 [file Data_Sheet_1.docx]

Table 1S. Some characteristics bacterial isolates

| **Tests** | ***Enterobacter cloacae* PMFKG-CV3** | ***Klebsiella oxytoca* PMFKG-CV4** | ***Serratia odorifera* PMFKG-CV7** |
| --- | --- | --- | --- |
| **Flagella** | + | - | + |
| **Motility test** | + | - | + |
| **DNAse** | - | - | + |
| **Adonitol** | - | - | + |
| **Meso-Erythritol** | - | - | - |
| **Mucate** | + | + | - |
| **Dulcitol** | - | + | - |

Table 2S. Identification tests for isolated bacteria (API 20E) and yeast (API 20C AUX strip, BioMérieux)

| **Tests** | ***Enterobacter cloacae***  **PMFKG-CV3** | ***Klebsiella oxytoca* PMFKG-CV4** | ***Serratia odorifera* PMFKG-CV7** | **Tests** | ***Saccharomyces cerevisiae***  **PMFKG-CV10** |
| --- | --- | --- | --- | --- | --- |
| [**ONPG**](https://microbeonline.com/onpg-test-galactosidase-principle-procedure-results/) | + | + | + | **GLU** | + |
| **ADH** | + | + | – | **GLY** | – |
| **LDC** | – | + | + | **2KG** | – |
| **ODC** | + | – | + | **ARA** | – |
| **CIT** | + | + | + | **XYL** | – |
| **H2S** | – | – | – | **ADO** | – |
| **URE** | – | + | + | **XLT** | – |
| **TDA** | – | + | + | **GAL** | + |
| **GEL** | – | – | + | **INO** | – |
| **GLU** | + | + | + | **SOR** | – |
| **MAN** | + | + | + | **MDG** | + |
| **INO** | – | + | + | **NAG** | – |
| **SOR** | + | + | + | **CEL** | – |
| **RHA** | + | + | + | **LAC** | – |
| **SAC** | + | + | + | **MAL** | + |
| **MEL** | + | + | + | **SAC** | + |
